# Optimized communication during risk disclosure to reduce nocebo headache after lumbar puncture—a study protocol for a randomized controlled clinical trial

**DOI:** 10.3389/fpsyg.2025.1521978

**Published:** 2025-02-26

**Authors:** Livia Asan, Johanna Sophie Gronen, Lorenz Peters, Christoph Kleinschnitz, Dagny Holle-Lee, Sven Benson, Ulrike Bingel

**Affiliations:** ^1^Department of Neurology, Center for Translational and Behavioral Neurosciences, University Hospital Essen, University of Duisburg-Essen, Essen, Germany; ^2^Institute for Medical Education, University Hospital Essen, University of Duisburg-Essen, Essen, Germany; ^3^Institute of Medical Psychology and Behavioral Immunobiology, Center for Translational and Behavioral Neurosciences, University Hospital Essen, University of Duisburg-Essen, Essen, Germany

**Keywords:** nocebo, expectation, study protocol, lumbar puncture, headache, communication, informed consent, side effect

## Abstract

Risk communication has been found to be a driver of nocebo effects in medical settings by enhancing negative expectations. In fact, merely disclosing the risk of side effects and complications of treatments or medical procedures increases reports of adverse events. Along these lines, it has been proposed that the occurrence of headache after lumbar puncture (LP), a routine diagnostic procedure in neurology, is caused to a large degree by the information delivered by the physician during the informed consent procedure. As withholding information conflicts with principles of patient autonomy, strategies are needed to mitigate nocebo-associated headaches without omitting the disclosure of risks. Here, we present a detailed study protocol for a preregistered, prospective, double-blind, randomized controlled clinical trial with N = 80 inpatients at the neurology department of the University Hospital Essen who have an indication for a diagnostic LP. The trial is designed to test whether optimized communication strategies aimed at minimizing nocebo effects during the informed consent procedure reduce headache-related impairment after LP compared to standard-of-care. Secondary outcomes include expectation of side effects, state anxiety, headache intensity and duration, use of on-demand pain medication, perceived warmth and competence of the physician, and satisfaction with the procedure.

## 1 Introduction

The nocebo effect refers to adverse outcomes that arise due to negative expectations. Classically, such effects are evident in adverse events reported in placebo arms of randomized controlled trials (RCTs), where adverse events in the placebo arm are often of similar quality and quantity to those in the verum arm, meaning that a large proportion of side effects are actually attributable to non-drug-related effects. For example, the nocebo effect was recently demonstrated in a systematic review and meta-analysis of large phase-III trials of COVID-19 vaccines (Haas et al., 2022a), in which 76% of reported systemic side effects were found to be unrelated to the actual receipt of the vaccine (Haas et al., 2022a). Subjective symptoms are particularly susceptible to the effects of negative expectations (Liccardi et al., 2004; Wolters et al., 2019). Headache, for instance, is one of the most commonly reported side effects in placebo arms of RCTs (Howick et al., 2018; Duncan et al., 2024). Nocebo-related headaches have also been observed following media coverage about health risks, with increased rates of headache after news reports about side effects from vaccinations against COVID-19 and human papillomavirus (HPV) as well after the reformulation of a thyroxine medication (Faasse et al., 2012, 2017; Asan et al., 2024). Moreover, nocebo-related headaches have also been confirmed in studies employing controlled test environments (Stovner et al., 2008; Benedetti et al., 2014). There is extensive empirical evidence demonstrating the powerful impact of the nocebo effect on health and wellbeing. Recent research has focused on both internal and external factors that contribute to the nocebo effect and has examined its neurobiological and neurochemical underpinnings (for recent reviews, see Colloca, 2024; Grosso et al., 2024).

The nocebo effect is well documented in placebo-controlled clinical trials and experimental work, but its relevance for clinical reality extends far beyond the application of inert treatments (Planes et al., 2016). For example, a study in healthy participants found that the analgesic effect of the highly potent opioid drug remifentanil on experimentally induced pain was almost entirely nullified when participants were told that the infusion of remifentanil had been stopped, despite the fact that it actually continued unchanged (Bingel et al., 2011). Such findings highlight the power of verbal suggestion as a potent driver of nocebo effects, in addition to prior negative experiences (Zunhammer et al., 2017). Communication by healthcare providers frequently conveys unintentional negative suggestions, increasing anxiety and arousal and facilitating the generation of nocebo effects in medical care (Lang et al., 2005; Vranceanu et al., 2012; Greville-Harris and Dieppe, 2015; Stortenbeker et al., 2018; Borelli et al., 2021). Critically, the mere act of informing a patient about risks and side effects of a provided treatment or procedure can itself increase the likelihood of a patient experiencing them. For instance, in a study on sexual dysfunction during finasteride treatment, 43.6% of the men who were informed about the rare occurrence of erectile dysfunction, decreased libido, and ejaculatory disorders reported experiencing these side effects, compared to only 15.3% of the patients who were not given this information (Mondaini et al., 2007).

Especially, when an invasive diagnostic procedure is planned, patients are routinely informed about relevant risks and potential complications before giving consent. The lumbar puncture (LP) is a particularly common diagnostic procedure in neurology, used to collect cerebrospinal fluid (CSF) from the spinal canal. Investigation of CSF is part of the basic workup of a plethora of neurological and neuropsychiatric symptoms and is often critical for the diagnosis of inflammatory processes of the nervous system (e.g., meningitis, encephalitis, multiple sclerosis, inflammatory neuropathies), subarachnoid hemorrhage, neurodegenerative diseases (e.g., Alzheimer’s disease), or oncological processes (e.g., meningeal carcinomatosis) (Jane and Wray, 2024). It involves inserting a needle into the subarachnoid space of the spinal canal at the lumbar level to passively drain CSF. A known potential side effect of this procedure is postdural puncture headache (PDPH) (Plewa and McAllister, 2024), which is thought to be caused by intracranial hypotension, resulting in strain on the meninges. Symptoms typically appear within 48–72 h and are usually, though not exclusively, orthostatic, that is, worsening when upright and improving when lying down (Plewa and McAllister, 2024). The incidence reported in the literature varies widely, ranging between 2 and 40%, but generally not exceeding 10% when atraumatic needles are used (Nath et al., 2018; Tumani et al., 2019; Plewa and McAllister, 2024). Given that lumbar puncture is a routine procedure, with thousands performed worldwide every day, any reduction of the burden of headaches due to nocebo effects is, therefore, likely to be impactful.

Back in 1981, Daniels and Sallie suggested that a significant proportion of headaches following lumbar puncture may be less related to the pathophysiology of PDPH and more likely to be nocebo-mediated, non-specific headaches (Daniels and Sallie, 1981). In their study of 28 patients, the risk of headache was disclosed to 15 patients but not to the remaining 13 patients. Upon assessing headache incidence at 4- and 24-h post-LP, seven (47%) of the informed participants reported headache, compared to only one person (8%) in the uninformed group. The results of this single study, with a small sample size and an ethically questionable approach, need to be interpreted with caution. However, given the known susceptibility of headaches to nocebo effects as well as the role of risk disclosure in promoting side effects, the potential for significant nocebo effects remains plausible. Importantly, this presents an ethical dilemma in medical practice: On one hand, informed consent is essential for preserving patient autonomy and must be upheld to ensure transparently informed consent and health-related decision-making. On the other hand, physicians are obligated to avoid potential harm from nocebo effects, in line with the principle of *primum non nocere*—first, do no harm.

A potential solution to this dilemma lies in the use of communication strategies that reduce negative expectations and minimize nocebo effects without obscuring risks or deceiving patients (Bingel and Placebo Competence, 2014). Several strategies targeting verbal and non-verbal communication have been successfully tested in experimental studies. *Positive attribute framing* of a risk entails stating the probability of *not* experiencing a side effect (Faasse et al., 2019). For example, this was demonstrated in a study on cybersickness in virtual reality, which found that participants who were told that “7 out of 10 people will not experience nausea” reported less nausea than participants who were told that “3 out of 10 people will experience nausea” and participants who were given general information about nausea occurrence (Mao et al., 2021). *Positive message framing* can be used to reframe the experience of a side effect as something positive and has been found, for example, to increase vaccine acceptance and reduce nocebo effects after vaccination against SARS-CoV2 (Bender et al., 2023; Crum et al., 2023). The use of *positive wording* and the avoidance of anxiety-laden terms can reduce perceived pain from needle injections (Varelmann et al., 2010). Furthermore, as discussions are frequently dominated by the possibility of adverse events, even if such events are uncommon, *emphasizing the value and goal* of a medical procedure helps to bring about a balanced risk–benefit assessment. Naming treatment benefits alongside risks has been shown to reduce nocebo weakness after insertion of a pain catheter (Zech et al., 2022). Moreover, goal setting is known to positively influence treatment adherence (Coppack et al., 2012) and health outcomes (Rief et al., 2017), while lack of clarity in treatment goals can lead to a less effective treatment (Geurtzen et al., 2020). *High warmth and empathy of the healthcare professional, conveyed through verbal and non-verbal cues,* lead to fewer reported side effects (Barnes et al., 2024), and warmth and competence positively influence outcome expectations (Seewald and Rief, 2024). One strategy to achieve positive doctor–patient communication is to *validate* patients’ experiences, which has been shown to be effective in attenuating nocebo effects (Greville-Harris and Dieppe, 2015). Finally, providing educational *information about the nocebo effect* resulted in reduced negative treatment expectations and fewer reported side effects (Pan et al., 2019; Michnevich et al., 2022; Gorner et al., 2024).

There are further promising strategies to attenuate the nocebo effect that have not yet been directly tested but have been shown to boost placebo effects or positively influence factors that, in turn, modulate nocebo effects (Bingel and Placebo Competence, 2014). For instance, research on attention and learning shows that information that is delivered first (*primacy effect*) and last (*recency effect*) is more easily retained in memory. Therefore, opening and closing an informed consent procedure (ICP) with positive aspects helps the patient to keep these aspects in mind for later consideration. Narrating from a *patient-centered perspective* rather than a doctor-centered perspective during medical consultation for a placebo with an alleged concentration-enhancing effect was found to lead to a higher intention to take the placebo (Haas et al., 2022b). Moreover, explicitly *addressing patients’ needs and specific concerns* during the consultation was shown to positively change patients’ attitudes toward participating in mammography screening (Fissler et al., 2015). Finally, *pointing out measures taken to prevent unwanted effects* can convey safety and competence, while *outlining effective coping strategies* that the patient can utilize in the case of adverse events empowers self-efficacy and reduces fear.

The goal of this study is to implement a protocol that encompasses optimized communication strategies to assess their effect on patient-reported headache-related impairment and overall burden after diagnostic lumbar puncture. Specifically, the study aims to test whether such communication can reduce nocebo-related symptoms without compromising the relevant content for informed consent. The publication of the detailed study protocol serves to improve reproducibility and transparency and to facilitate the translation of these strategies for use in clinical practice.

The study was designed as a preregistered, prospective, randomized controlled trial conducted at the University Hospital Essen. Between December 2023 and May 2024, inpatients scheduled for a diagnostic lumbar puncture (with no prior lumbar puncture experience and for diagnostic purposes only) were enrolled and randomly assigned to either the optimized communication group (OPT) or the standard-of-care group (SOC), which serves as the control group, to reach the target of N = 80 patients (n = 40 per group). In the OPT group, optimized communication strategies were employed to reduce nocebo effects, while the SOC group received standard information. The same physician (LA) performed all informed consent procedures and lumbar punctures, and data were collected through online surveys and interviews conducted by a study assistant (JSG) who was blinded to group allocation. The primary outcome is the level of headache-related impairment, measured on a numerical rating scale (0–10) at 3 h, 24 h, and 72 h postprocedure.

## 2 Methods and analysis

### 2.1 Trial design

This prospective study was conducted using a parallel-group design with two groups. In the experimental group, the optimized communication protocol was applied (OPT), whereas the standard-of-care group (SOC) received communication according to a control protocol that uses standard communication observed in the clinic with no particular consideration of the effects of communication. The required sample size for the planned analysis of the main outcome (i.e., complete datasets from t1 to t5) was N = 80 inpatients, recruited in the general wards of the Neurology Department at the University Hospital Essen, with a balanced 1:1 group allocation ratio (n = 40 for SOC, n = 40 for OPT). The study was preregistered at the German Clinical Trials Register (DRKS00032272). This study protocol aligns with the SPIRIT guidelines (Standard Protocol Items: Recommendations for Interventional Trials) and follows the SPIRIT-PRO extension for patient-reported outcomes, as specified in Supplementary Table S1 (Chan et al., 2013; Calvert et al., 2018).

### 2.2 Ethics statement

The study protocol was approved by the Ethics Committee of the University Hospital Essen (22-11020-BO) on June 14, 2023.

### 2.3 Inclusion criteria

Any adult (minimum 18 years old) with a medical indication for an elective, that is, planned lumbar puncture for diagnostic purposes during an inpatient stay on a general ward in the Neurology Department at the University Hospital Essen. The indication as well as the absence of contraindications for LP were confirmed by the ward physicians and by the study physician.

### 2.4 Exclusion criteria

Patients were not eligible if one or more of the following criteria were met: presence of contraindications against LP, as defined in guidelines (Engelborghs et al., 2017; Tumani et al., 2019), which include elevated intracranial pressure, infection at the puncture site, use of anticoagulant medications, coagulopathy, or thrombocytopenia; emergency indication for lumbar puncture; insufficient mental capacity to provide informed consent; impaired consciousness; limited ability to complete surveys on an electronic tablet; intended therapeutic purpose of lumbar puncture (i.e., spinal tap for suspected intracranial idiopathic hypertension or normal pressure hydrocephalus); history of prior lumbar puncture (or failed attempts); extreme scoliosis, extreme obesity with BMI > 50, or other medical conditions likely to complicate lumbar puncture; indication for performing lumbar puncture under fluoroscopy; presence of a chronic headache condition according to the International Classification of Headache Disorders (ICHD)-3 criteria and/or 15 or more headache days in the last 30 days; headache as the primary complaint leading to admission or LP indication.

### 2.5 Criteria for discontinuing study participation

Study participation was to be terminated preemptively if the following occurred after enrollment: LP no longer indicated by medical personnel or the patient declined or aborted the procedure; failed attempt at lumbar puncture with no collection of CSF; patient retroactively withdrew consent for study participation or for use of the data; other medical or logistical barriers to study continuation on the part of the patient (e.g., discharge before the LP, significant deterioration in health, extended unavailability or incompliance of the patient for study participation) or the study personnel (e.g., unplanned unavailability of study personnel without adequate replacement or feasibility of rescheduling the lumbar puncture); later emergence of exclusion criteria (i.e., change in LP indication to a therapeutic CSF drainage). Patients who did not complete study time point t5 (3 days after LP) for any of these reasons will not be included in the final per-protocol analyses.

### 2.6 Patient screening and enrollment

The blinded study assistant (JSG) recruited inpatients from the three general wards at our neurology department. First, inpatients were screened for an indication for a diagnostic LP as determined by the treating ward physicians. Inclusion and exclusion criteria were then checked, and all eligible patients were approached and informed about the option to participate and gave written informed consent for enrollment.

Patients were informed that all eligible patients undergoing lumbar punctures at our facility were currently being asked to participate in a study to evaluate the entire process and monitor the quality of care and that they would be asked to complete questionnaires at different time points before and after the LP for this purpose. We did not disclose the fact that the informed consent procedure (ICP) would be the object of the intervention and that headache as a side effect was the main outcome, as this would elicit attention to the symptom and lead to reporting bias. Instead, we stated that the study would test the implementation of a new protocol for the preparation and postprocedure management of lumbar punctures on the ward by the physician and compare this to the usual routine. Patients were told that they would be randomly assigned to one of two groups. It was emphasized that no technical aspect of the execution of the LP would be altered, irrespective of whether or not they participated in the study. In the written participant information, patients were made aware that in some studies, patients may not be fully informed about all objectives and contents of the investigation for scientific-methodological reasons, but that compliance with ethical standards is always maintained. All participants provided written informed consent before data collection.

### 2.7 Sample size, randomization, and blinding

The sample size of N = 80 for the final analysis was chosen for reasons of feasibility while ensuring sufficient power to detect a clinically meaningful effect. With a power of 0.90, the study can detect an effect size f of at least 0.16 for the interaction effect between the group and time point on the primary outcome measure (headache-related impairment) (G*Power 3.1.9.7). This corresponds to a small to medium effect size. The recruitment period was set at 6 months (December 2023–May 2024), based on predetermined staff resources and estimates of the expected number of eligible study patients from recruitment from the wards. This timeline was expected to yield an effective sample size of 80 participants, accounting for an anticipated attrition rate of 15%.

Group allocation was performed after enrollment (i.e., after patients had provided written informed consent) via clustered block randomization, with a block consisting of 10 sequentially enrolled patients. To minimize the risk of communication between participants potentially influencing the outcomes, which can be a concern in settings like inpatient stays, the entire block was assigned to the same group. The sequence of all blocks was randomized before the start of the study based on a randomization list created in R. The last block of each group was truncated and ended as soon as the targeted sample size of n = 40 per group (not counting dropouts) was reached. The randomization list and the mode of randomization (clustered block randomization) were concealed from the blinded study assistant, who was responsible for recruiting and data collection, as well as from the clinical staff, to avoid unintended indirect influences from nurses or treating ward physicians. Only the study physician performing the ICP and LP according to the group allocation had the randomization list and was unblinded to group allocation.

### 2.8 Protocol for standard-of-care and optimized communication

The protocols for the ICP differed between the two groups in terms of specific and predefined aspects of verbal and non-verbal communication.

First and foremost, the study physician adhered to the group-specific guiding script when orally presenting information about the indication, instructions, steps of the procedure, risks, and recommendations in the ICP. The SOC condition intended to reflect real-world clinical communication, characterized by negatively framed statements and a paucity of patient-centered interaction behaviors (Lang et al., 2005; Tackett et al., 2013; Sharp et al., 2022). Table 1 lists key elements of the group-specific guiding scripts and the communication technique in OPT. The entire guiding script for each group is provided in Supplementary Text S1. If questions from the patients arose, the physician followed predefined rules for interactions and demeanor toward the patient. The physician’s body language also differed between the two groups. In the control group, the physician stood at the patient’s bedside with arms mostly crossed, not seeking eye contact, and checking the time on their cellphone two or three times. The optimized communication protocol involved the physician sitting on a chair next to the patient, maintaining eye contact, using open gestures, and modulating their tone of voice and facial expressions to convey empathy where appropriate. Supplementary Videos S1, S2 give an impression of the informed consent procedure according to SOC and OPT in English.

**Table 1:** Key elements of the verbal information during the informed consent procedure for the standard-of-care group (SOC) and the optimized communication group (OPT).

	Standard-of-care (SOC)	Optimized communication (OPT)	*Communication strategy used*
Indication	“We need the spinal fluid to proceed with your case.”	“We’re investigating the spinal fluid to better understand how we can best help you.”	*Primacy effect, goal orientation, patient-centered narration*
	–	“We perform these spinal taps very often here. For me, it’s like drawing blood.”	*Primacy effect*
	“There is not a good alternative to investigating the spinal fluid, so unfortunately, if we want to know these results, there’s no way around it.”	“The spinal fluid is especially helpful for understanding what’s going on in the nervous system. That’s why we do it so often.”	*Positive message framing, goal orientation*
Procedural information	“You’ll sit there and arch your back. You need to tuck your chin into your chest. It’s important that you stay in this position because otherwise, it will be more difficult for me to get the needle between the spinous processes of the vertebrae into the spinal canal.”	“You’ll sit on the edge of the bed and arch your back. You can see an illustration of it here. It’s important to tuck your chin into your chest as this is the ideal position. That will already be a big help for me.”	*Positive message framing, goal orientation*
	“As you can see, here in the spinal canal, where we want to go, is also the spinal cord. We want to avoid getting close to that at all cost. That’s why I will insert the needle lower down here.”	“Here in the spinal canal there is also the spinal fluid. Sometimes patients ask me if the spinal cord is not close to that site. But I can reassure you, as that ends higher up and we are at a safe distance; our target area is down here.”	*Addressing common concerns, reassurance, positive wording*
	“The tip of the needle may touch nerve fibers, which you might feel as a brief electric shock in your leg.”	“It’s possible that we might tickle the nerve fibers during the procedure, which you might feel as a brief tingling in the leg. But when that happens, we know we are in the right spot, and we’ll already be done after a few minutes.”	*Positive wording, positive message framing*
Preamble before risk disclosure	“Unfortunately, complications cannot be completely ruled out. On this form here, various risks are listed. I will mention some of them to you.”	“As a doctor, it’s my duty to inform you about the risks of the procedure.”	*Positive wording, patient-centered narration*
	–	“However, side effects are rare, and the vast majority of patients do not notice anything afterward. You should also know that sometimes side effects occur simply because you are informed about them. Just knowing about potential side effects can alter your body’s perception; it might have happened to you before, you listen to yourself a bit more and become more aware of small changes that you would not otherwise notice. This phenomenon is called the nocebo effect, and it has been documented in many scientific studies. I can give you an example from my own experience: I was at a family gathering, and it later turned out that my nephews and nieces had headlice. You cannot imagine how much my head and everywhere else itched when I heard that, even though I did not have any lice myself in the end. So, my advice: Keep this in mind whenever you hear about side effects. It’s best to distract yourself after the procedure, perhaps by reading something.”	*Nocebo information*
Risk disclosure	“Infections, for example, are always possible when a needle is inserted into the body, as germs could potentially be introduced.”	“I will work carefully and in sterile conditions to minimize the risk of infection.”	*Stressing prevention measures*
	“Also, in principle, bleeding or tissue injury from the needle can occur, but this is very unlikely.”	“This is the most common procedure in neurology; we perform it several times a day at this university hospital, and I have never encountered a relevant bleed or injury caused by it.”	*Reassurance*
	“We will drain some spinal fluid from the body. The most common side effect that could arise from this is headache. Therefore, you might get a headache, which is worse when you are upright, so sitting or standing.”	“The body quickly replenishes the removed spinal fluid. Sometimes, patients may experience headaches, which improve significantly when lying down.”	*Positive wording*
	“Such headaches occur in about 10 percent of cases after a lumbar puncture.”	“However, the vast majority of patients, about 9 out of 10, do not experience this at all.”	*Positive attribute framing*
Recommendations	“If headaches occur, you should lie flat and drink plenty of fluids, and if necessary, you can get painkillers.”	“If you do notice any such discomfort, you can help it pass quickly by lying flat and drinking plenty of fluids. We can also support you with effective pain medication if necessary.”	*Positive wording, stressing coping mechanisms*
	“We recommend that you lie flat for one hour after the puncture and drink plenty of fluids. However, this is not strict bed rest.”	“We recommend that you lie flat for one hour after the procedure and drink plenty of fluids. However, this is not strict bed rest.”	*- (kept equal to avoid influence on the behavior)*
Results	“The individual results of the examination will be communicated to your doctor, who will share them with you.”	“You’ll most likely want to know when you’ll get the results—Dr. XYZ will discuss them with you as soon as they come back from the lab.”	*Patient-centered view, addressing patient needs*
Closing	“We can perform the lumbar puncture today; I’ll be able to squeeze it into my schedule, so you’ll get it over with quickly. I assume you do not want to take 24 h to think it over before we start the lumbar puncture?”	“We can perform the puncture today, so you’ll have the results quickly. Therefore, we’d like to offer to do this today.”	*Goal orientation, positive wording*
	–	“Overall, it is a safe and very commonly performed routine procedure.”	*Recency effect*

For all patients, the ICP and the LP were solely performed by one female study physician (LA) with established expertise in performing lumbar punctures (~100 performed). At the time of the ICP and LP, she had been a resident in neurology for 3.5 years and was 30 years old. She is White and speaks German as her native language.

The ICP took place in the patient’s room. In both groups, the physician displayed a standard information printout commonly used for ICP (Thieme Compliance GmbH). While giving the instructions for the patient’s positioning and describing the process of the lumbar puncture, the physician demonstrated the required position and the steps of the LP procedure with a few drawings on an illustration on the printout. When a risk was mentioned, the respective part in the text stating this risk was circled with a pen. The physician indicated the approximate time when the LP was to be performed, and both the physician and the patient signed the leaflet to confirm informed consent. The physician offered to copy the signed information leaflet for the patient to keep.

If the patient expressed concerns or worries at any time during the ICP or LP, the physician used scripted reactions according to the group allocation:

SOC: “You do not need to be afraid. It will not take long. It’s best if we get it over with quickly. Then you’ll have the worst part behind you.”

OPT: “I can understand if you are feeling a bit uneasy about this. I would probably feel the same way in your position. But, you know, most patients feel relieved afterward and are surprised at how well it went.” *(emotional validation, positive wording).*

The insertion of the needle tip was announced in the groups as follows:

SOC: “Caution, here comes the prick.”

OPT: “Alright, I am starting now.” *(positive wording).*

### 2.9 Lumbar puncture

Lumbar puncture was performed according to the usual routine in our department. Only atraumatic systems consisting of a facet-tip introducer and an atraumatic Sprotte needle were used (PAJUNK Sprotte Lumbar). In total, 22-, 21-, or 20-gauge needles with lengths of either 90 or 120 mm were used at the physician’s discretion, based on the patient’s physique. To enable a comparison between groups, the needle size and length used, number of attempts until successful puncture of the subarachnoid space, milliliters of CSF collected, and total time spent at the patient’s bedside for LP were noted. Inpatient roommates and visitors were asked to leave the room for the duration of the procedure.

The patient was asked to sit at the edge of the bed and the bed was elevated. To ensure a stable and comfortable position, the patient’s feet rested on a chair. The patient was asked to adjust his/her position, with the chin tucked into the chest and the upper body held in a kyphotic posture. The physician disinfected her hands before any contact with the patient environment and put on non-sterile gloves and a face mask. The horizontal line between the palpable tops of the iliac crest on both sides indicates the height of the spinous process of lumbar vertebra L4. The interspinous space of lumbar vertebrae L3-L4 or L4-L5 was chosen for needle insertion. Diligent repeated, spacious disinfection of the skin (Kodan) was performed. A sterile working field with sterile swabs, adhesive bandages, and needles was prepared, and the physician disinfected her hands again and switched to sterile gloves. The insertion of the introducer was announced to the patient according to the group-specific wording. The Sprotte needle was then inserted through the introducer to enter the subarachnoid space. Upon loss of resistance (indicating penetration of the Dura), the metal stylet within the Sprotte needle was retracted to allow backflow of CSF. A target amount of approximately 10 mL of CSF was collected before inserting the stylet again and withdrawing the needle, and the patient was told that the procedure was finished, and he/she could sit up normally. A small adhesive bandage was applied at the puncture site. The patient was briefly reminded about the recommendation to rest in a supine position. The physician then left the room to take care of the processing of the CSF samples.

### 2.10 Main outcome

#### 2.10.1 Headache-related impairment

The main outcome is headache-related impairment, and was rated 3 h (t3), 24 h (t4), and 72 h (t5) after a lumbar puncture on a numerical rating scale (NRS) with integer values ranging from 0 (= no impairment) to 10 (maximum imaginable impairment). Patients were asked to rate their headache-related impairment since the lumbar puncture (t3) or since the last survey time point (t4, t5), respectively: “How severely have you felt impaired by headache since the lumbar puncture?” (original in German: “Wie stark fühlten Sie sich seit der Lumbalpunktion durch Kopfschmerzen beeinträchtigt?”). The construct of impairment was chosen as it allows/encourages the patient to integrate different dimensions of the individual negative pain experience (i.e., impact on activity, pain unpleasantness, pain intensity, duration) into his/her assessment.

### 2.11 Secondary outcomes

Secondary outcomes are intended to provide supporting evidence for the positive effects of the intervention and explore potential modulating effects on the main outcome.

#### 2.11.1 Expectation of unwanted effects

As part of an assessment of procedure-related expectations using modified items of the Treatment Expectation Questionnaire (TEX-Q) (Shedden-Mora et al., 2023), the expectation regarding the extent of unwanted side effects after LP was rated on an NRS from 0 to 10 at t2 (immediately after ICP). The precise wording of the modified questions is provided in Supplementary Text S2.

#### 2.11.2 State anxiety

State anxiety was assessed immediately after ICP (t2) by calculating the total score from the 20 items of the state anxiety subscale from the State–Trait Anxiety Inventory (STAI) (Ferreira and Murray, 1983).

#### 2.11.3 Headache incidence

Patients were asked if they had experienced any headache (binary yes/no response) since LP (t3) or since the last survey (t4, t5). Headache incidence describes the number of patients who experienced headache (= yes response) at any of these time points, divided by the number of all patients in the respective group.

#### 2.11.4 Headache intensity and duration

Current as well as mean and maximum headache pain intensity (NRS 0–10) as well as headache duration (in hours) since LP (t3) or since the last survey (t4, t5) were assessed.

#### 2.11.5 Use of on-demand pain medication

The use of on-demand pain medication (binary yes/no response) since LP (t3) or since last survey (t4, t5) was assessed.

#### 2.11.6 Perceived warmth and competence of the physician

At t2, the perceived warmth and competence of the physician were assessed using 12 items rated on a 5-point scale (1 = not at all to 5 = extremely) (Seewald and Rief, 2023). The “warmth” subscale score was calculated as the mean of items 1–6, while the “competence” subscale score was calculated as the mean of items 7–12.

#### 2.11.7 Satisfaction with the procedure

Patients were asked to rate their overall satisfaction with the procedure 3 h after LP (t3) based on the German school grading system from 1 to 6, where 1 is the best grade and 6 is the worst grade.

### 2.12 Study schedule and data collection

**Figure 1:**
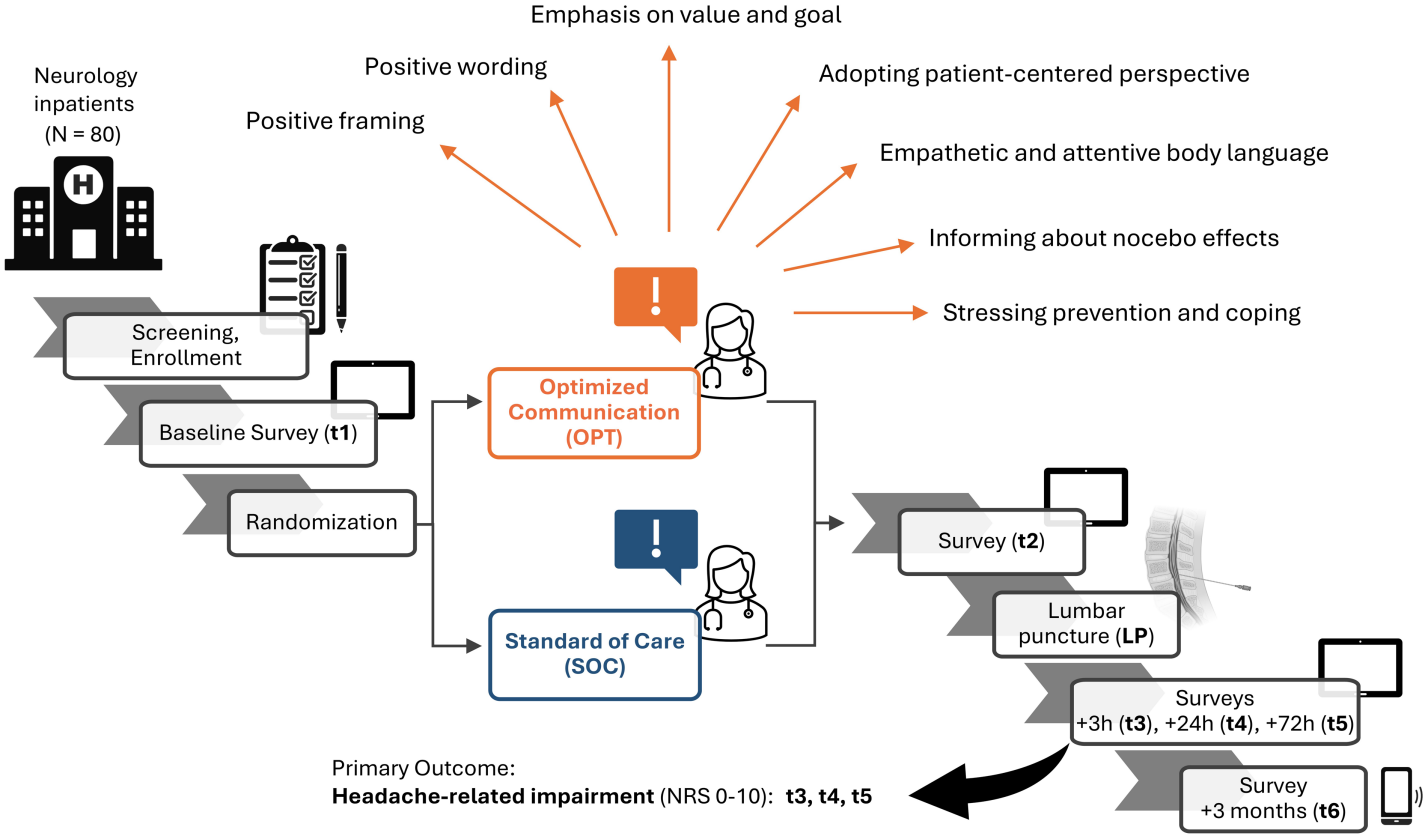
Study schedule at a glance. Inpatients in the neurology department of the University Hospital Essen requiring a diagnostic lumbar puncture (LP) were recruited to reach the target of N = 80. Screening and enrollment with obtaining consent were followed by the baseline survey (t1). Patients were randomized (n = 40 per group) to either the group receiving informed consent procedure (ICP) with the standard-of-care (SOC, blue), or optimized communication strategies (OPT, orange), some of which are displayed (orange arrows). A second survey follows after ICP (t2). The LP is performed, and surveys follow at 3 h (t3), 24 h (t4), and 72 h (t5) after LP. The completion of t5 marks the end of the main investigation period, in which the primary outcome—headache-related impairment on a numeric rating scale (NRS) from 0 to 10—is assessed. A follow-up online survey and telephone interview are performed 3 months after LP (t6). Created in BioRender. (Asan, 2025).

Figure 1 displays an overview of the study schedule. The main investigation period spanned from inclusion to 72 h (i.e., 3 days) after LP. A follow-up survey was conducted 3 months after LP. After enrollment, participants were assigned a unique pseudonym using the software ALIIAS (Englert et al., 2023). ALIIAS is equipped with integration to LimeSurvey (LimeSurvey GmbH, Hamburg, Germany), an open-source web server-based survey system, which was used to collect all digital survey data in this study (Klieve et al., 2010). Each survey time point consisted of a digital survey, which the patient completed alone without any assistance, as well as a subsequent interview by the blinded study assistant, who recorded the answers in a separate online survey. Only at survey time point t2 (directly after the doctor’s explanation of the LP) did the patient exclusively complete an online survey without a subsequent interview. Table 2 summarizes the collected data and the questionnaires used.

**Table 2:** Overview of collected data and surveyed questionnaires.

Time point	Data entered directly by the patient (web-based survey)	Data provided by interview and entered by blinded study assistant
t1—Baseline assessments	Trait anxiety and depression (STADI-Trait) Baseline generic symptoms (modified GASE baseline) during past 7 days Current headache intensity	Demographic and biometric characteristics Medication BP and HR at admission Alcohol consumption Pack years Neurological symptoms Knowledge of (suspected) diagnosis Illness perception (modified B-IPQ) Previous experiences with similar procedures From physician: Reason for admission, LP indication, and (suspected) diagnosis
ICP (informed consent procedure)		Duration of physician contact during consent Copy of print ICP leaflet requested Transcript of audio recording
t2—after ICP	Treatment expectations (**modified TEX-Q**) State anxiety (**STAI-state**)	
LP (lumbar puncture)		Duration of physician contact during LP Anxiety before needle insertion 0-10 Needle insertion pain Amount of CSF collected in ml Needle bore size in gauge (G), needle length in mm Count of attempts
t3—3 h after LP	Current pain at needle insertion site Congruence with expectation Generic symptoms and LP side-effect attribution (modified GASE) **Current headache intensity** **Headache-related impairment since LP** **Duration of headache since LP (in hours)** **Minimum, maximum, and mean headache intensity since LP** Usage of the print ICP leaflet **Warmth and competence (W&C)** **Overall satisfaction with procedure**	Fluid intake in liters since LP Duration of bedrest in minutes If headache was present: Headache features (quality, localization, accompanying symptoms, restriction of physical activity) **Request for on-demand pain medication**
t4—24 h after LP	Generic symptoms and LP side-effect attribution (modified GASE) **Current headache intensity** **Headache-related impairment since t3** **Duration of headache since t3 (in hours)** **Minimum, maximum, and mean headache intensity since t3**	If headache was present: Headache features (quality, localization, accompanying symptoms, restriction of physical activity) **Request for on-demand pain medication**
t5—72 h after LP	Generic symptoms and LP side-effect attribution (modified GASE) **Current headache intensity** **Headache-related impairment since t4** **Duration of headache since t4 (in hours)** **Minimum, maximum, and mean headache intensity since t4** Belief about group allocation	If headache was present: Headache features (quality, localization, accompanying symptoms, restriction of physical activity) **Request for on-demand pain medication**
t6—3 months after LP	Generic symptoms (modified GASE) during past seven days Current headache intensity Headache-related impairment since LP Minimum, maximum, and mean headache intensity since LP Overall satisfaction with procedure Belief about group allocation Belief about study goal	Knowledge of (suspected) diagnosis after inpatient stay Illness perception (modified B-IPQ) Pre-existence of diagnosis of primary headache Average number of headache days and days with headache pain medication per month in the three months before and after LP Occurrence of new persistent headache since LP Headache features (quality, localization, duration of attacks, accompanying symptoms, restriction of physical activity) From physician and medical records: confirmation of diagnosis/suspicion

At all survey time points (t1–t6), a blinded study assistant collected patients’ data on the electronic study tablet. All data until t3 were collected in the hospital, while t4 and t5 were collected via an online survey and telephone interview if the patient had already been discharged. During the informed consent procedure (ICP) and lumbar puncture (LP), data were collected by the unblinded study physician. Assessments of the main (bold and underlined) and secondary outcomes (bold) are highlighted.

B-IPQ, Brief Illness Perception Questionnaire (Broadbent et al., 2006); GASE, General Assessment of Side Effects (Rief et al., 2011); ICP, Informed consent procedure; LP, Lumbar puncture; STADI-Trait, trait subscale of the State–Trait Anxiety and Depression Inventory (Laux et al., 2013); STAI-state, state subscale of the State–Trait Anxiety Inventory (Ferreira and Murray, 1983; Laux et al., 2013); TEX-Q, Treatment Expectation Questionnaire (Shedden-Mora et al., 2023); W&C, warmth and competence of the physician (Seewald and Rief, 2023); BP, blood pressure; HR, heart rate.

From inclusion to the survey time point 3 h after the lumbar puncture, the study took place exclusively during the inpatient stay in the clinic using the electronic study tablet and in direct personal conversation with the blinded study assistant. All other time points either occurred during the inpatient stay or after the patient’s discharge; in the latter case, the surveys were completed on the patient’s own electronic device and the interview was conducted by telephone.

The main and secondary outcomes were assessed at different time points, as described above. Since this study is part of a larger collaborative research effort investigating treatment expectations and potential modulators in different settings,^1^ participants were also asked to complete additional psychological questionnaires and provide biometric, demographic, and health data.

#### 2.12.1 Survey time point 1 (t1)—baseline assessments

After enrollment, patients completed questionnaires to assess trait anxiety and depression [trait subscale of the State–Trait Anxiety and Depression Inventory, STADI-Trait (Laux et al., 2013)] and the baseline presence and severity of the following symptoms during the past 7 days: nausea, headache, vertigo, back pain, changes of vision, hearing or sensation, and photo- or phonophobia symptoms [modified General Assessment of Side Effects, GASE (Rief et al., 2011)]. Current headache intensity was assessed on a numerical rating scale (NRS) from 0 to 10. Basic demographic and biometric characteristics were recorded (age, sex, education, height and weight, blood pressure, and heart rate at admission) as well as current medication, days with alcohol consumption per month, and nicotine consumption (in pack years). The reason for admission was stated freely (e.g., neurological symptoms or recent findings requiring further investigation). The presence of a suspected or known underlying neurological diagnosis was also recorded. Illness perception was assessed using the following three items from the Brief Illness Perception Questionnaire (modified B-IPQ) (Broadbent et al., 2006): experience of symptoms, impact on daily life, and degree of concern. Information was further obtained on patients’ prior experiences with similar procedures, that is, epidural or spinal anesthesia, and their satisfaction with these procedures. Finally, the reason for admission, indication for LP, and suspected diagnosis as given by the treating ward physicians were noted.

#### 2.12.2 Informed consent procedure (ICP)

The ICP was performed according to the group-specific protocol (see Supplementary Text S1). It was registered whether or not the patient requested a copy of the signed information printout, and the time spent with the patient for the ICP was noted. The informed consent discussion was audio-recorded using a small recorder in the physician’s lab coat; the recording began just before the physician entered the room and was terminated directly after she left the room. The audio recording was later transcribed to control for adherence to the protocols, and any patient-identifying contents were removed.

#### 2.12.3 Survey time point 2 (t2)—directly after ICP

After completion of the ICP, the study physician informed the study assistant that the patient could now be approached for the t2 survey. Although there was no prespecified delay between the ICP and t2, the study physician waited for several minutes to ensure that the study assistant could not know the true duration of the ICP and was, therefore, not at risk of being unblinded. The survey at t2 only included questionnaires that the patients completed on the tablet by themselves. Five items from the Treatment Expectation Questionnaire were modified to fit the purposes of the study (modified TEX-Q) (Shedden-Mora et al., 2023). These items measured the extent to which patients regarded the procedure as being associated with expected: (1) burden, (2) undesirable effects, (3) pleasant course, (4) satisfaction with the procedure, and (5) personal responsibility for its success. The state subscale of the State–Trait Anxiety Inventory (STAI-state) includes 20 items and was used to assess state anxiety after ICP (Ferreira and Murray, 1983; Laux et al., 2013).

#### 2.12.4 Lumbar puncture (LP)

The patient was approached in his/her hospital room for the LP. Once the patient had adopted the required position and the physician had thoroughly disinfected the skin area, prepared a sterile field, and was just putting on sterile gloves, the patient was asked to rate how nervous he/she currently felt about the imminent LP on an NRS from 0 (not at all) to 10 (worst imaginable nervousness). Upon successful puncture (indicated by CSF backflow), the patient was asked to rate the pain during needle insertion from 0 (no pain at all) to 10 (worst imaginable pain). After completion of the procedure, the physician left the room and immediately noted the number of necessary needle insertions into the skin until successful CSF drainage, the size and length of the needle used, and the total number of milliliters of CSF collected. The total contact time between physician and patient (from the physician entering to leaving the room) was noted.

#### 2.12.5 Survey time point 3 (t3)—3 h after LP

Three hours after completion of the LP, the patient was asked to complete the next survey on the tablet. The survey asked about current pain in the needle insertion site (NRS 0–10), congruence with the patient’s expectation about the course of the procedure (yes, as expected/better than expected/worse than expected), and generic symptoms and their attribution to the LP using the modified GASE (nausea, headache, dizziness, back pain, visual disturbances, hearing disturbances, abnormal sensations, phono- or photophobia, or other complaints specified by the patient). Current headache intensity (NRS 0–10), headache-related impairment since LP, duration of headache (in minutes), and minimum, maximum, and mean headache intensity since LP were further assessed (NRS 0–10). If patients had requested a print copy of the ICP leaflet, they were asked to indicate whether they had read it (no/partially/entirely) and if so, whether they found it useful (yes/no). Warmth and competence of the physician were evaluated and general satisfaction with the procedure was rated (Seewald and Rief, 2023). To account for potential behavioral influences on headache after LP, the amount of fluid intake and the duration of bed rest since LP were noted. If patients reported headache, the interviewer structurally assessed headache history in more detail in terms of quality, localization, accompanying symptoms (features of migraine or trigeminal-autonomic headache), and exacerbating factors (physical activity, orthostatic features) to check whether the headache could be assigned to a specific headache disorder such as PDPH or migraine, as characterized by the International Classification of Headache Disorders (ICHD-3). Finally, the utilization and effectiveness of on-demand pain medication (against headache or other pain) since LP were assessed.

#### 2.12.6 Survey time point 4 (t4)—24 h after LP

The patient was approached for t4 24 h after completion of the LP. As at t3, generic and specific side effects since the last survey (t3) were assessed using the modified GASE. Similarly, headache-related impairment since t3, and if applicable, duration, minimum, maximum, and mean headache were noted, and headache features were recorded. Again, utilization and effectiveness of on-demand pain medication were noted.

#### 2.12.7 Survey time point 5 (t5)—72 h after LP

T5 took place 72 h after completion of the LP and contained the same questions as at t4, relating to the time since the last survey. Patients were also asked to guess the group they believed they had been assigned to (application of the current standard procedure vs. application of the new test protocol) through one additional question in the tablet-based survey.

#### 2.12.8 Survey time point 6 (t6)—3 months after LP

A final online survey and telephone interview were conducted 3 months (target: 12 weeks = 84 days) after LP as a follow-up. The online survey assessed generic symptoms in the past 7 days and their attribution to the LP (modified GASE), the overall satisfaction with the LP, current headache intensity, headache-related impairment since LP, and minimum, maximum, and mean headache intensity. Participants’ beliefs about their study group allocation and the study’s overall goal were also queried. In the telephone interview, patients’ knowledge of their diagnosis and their illness perception were assessed again (modified B-IPQ). Finally, patients were asked whether they had a known diagnosis of primary headache and whether the number of days with headache and of days requiring pain medication due to headache per month had changed between the 3 months before and the 3 months after LP. Specifically, the occurrence of a new headache that persisted and never faded for at least 15 days since LP was queried. Headache characteristics were assessed again to determine their alignment with a specific headache disorder.

### 2.13 Data management

In addition to the participant consent and the participant list, personal data are stored exclusively in pseudonymized form. Pseudonymized survey data collected using LimeSurvey is exported and stored on the University Medicine Essen Cloud, and a copy on an external hard drive is securely deposited and locked in the facilities of the Department for Clinical Neurosciences and Translational Pain Research at Essen University Hospital. The pseudonymized data will also be analyzed within the framework of the Collaborative Research Center SFB 289 on “treatment expectation” (see text footnote 1, central project lead by senior author Ulrike Bingel) provided that the patient has given separate consent for this.

The paper-based participant consent forms and sheets that contain the full names and contact details of the participants will be stored separately at the study site for 10 years and will not be transferred into electronic form. We are committed to sharing anonymized raw data alongside analysis codes from all results presented in future publications from this study.

### 2.14 Analysis plan

Data will be analyzed in R (R Core Team).^2^

#### 2.14.1 Main outcome: headache-related impairment

We hypothesize that headache-related impairment (main outcome) will be lower in the OPT group than in the SOC group during the main investigation period, assessed at three time points: 3 h (t3), 24 h (t4), and 72 h (t5) after LP. To test this hypothesis using a 2×3 mixed factorial study design, we will apply two-way repeated measures analysis of variance (ANOVA) to calculate an F-statistic on the main effect of the factor group (OPT vs. SOC) as well as the interaction effect between group and time point. The significance level (alpha level, type II error rate) for the probability over F (Pr(>F), *p*-value) will be set at 0.05. If *p* < 0.05, *post hoc* contrasts with correction for multiple comparisons (Bonferroni–Holm) will be performed. If the data do not meet assumptions of normality and homoscedasticity, we will instead use aligned rank transform ANOVA as the non-parametric alternative for the assessment of main effects and interactions (Wobbrock et al., 2011). The study will follow a per-protocol approach for the analysis.

#### 2.14.2 Secondary outcomes

Current, mean, and maximum headache intensity will be tested using two-way repeated measures ANOVA or the non-parametric alternative, in alignment with the analysis of the main outcome. The headache incidence and the use of on-demand pain medication will be used to calculate odds ratios to compare the odds of developing headaches or using pain medication up until 72 h after LP between the OPT and SOC groups. Differences in expectations regarding the extent of unwanted side effects, state anxiety, perceived warmth and competence of the physician, and satisfaction with the procedure will be assessed using independent t-tests or Wilcoxon rank-sum tests (in the case of non-parametric data). For confirmatory analyses of group differences in the secondary outcomes, an alpha level of 0.05 after comparison for multiple testing using the Bonferroni–Holm procedure will be regarded as statistically significant.

#### 2.14.3 Exploratory analyses

The number needed to treat with optimized communication to prevent a certain level of headache-related impairment can be assessed exploratively (e.g., greater than 2 out of 10). Analyzing differences in reported side effects other than headache (assessed in the modified GASE questionnaire) allows us to test whether the optimized communication strategy influenced a wider range of side effect symptoms. Furthermore, the dataset lends itself to the exploration of associations between the study’s outcomes and demographic as well as baseline psychometric characteristics (e.g., age, sex, trait anxiety, trait depression), as their contribution to the outcomes can be controlled for and potential influences between outcomes, that is, through modulation, can be explored. The importance of perceived warmth and empathy of the physician—largely influenced by non-verbal communication—will be examined by analyzing the variance in side effect outcomes explained by patients’ ratings of the physician’s warmth, also probing for a potential modulatory influence on the group effect. The exploratory analyses are intended to make novel discoveries and help generate new hypotheses regarding biopsychosocial mechanisms through which the optimized communication might act or regarding predictive factors that might identify patients who benefit most from our optimized communication protocol.

## 3 Discussion

This study investigates the cumulative impact of several communication strategies designed to reduce nocebo-related headaches after lumbar puncture. Our objective is to apply knowledge derived from preclinical research on expectation effects and their modifiers, testing the efficacy of easily implementable techniques to diminish the nocebo component of procedure-related unwanted effects in a naturalistic clinical setting.

The nocebo effect is arguably more prevalent and impactful in clinical settings than its better-studied positive counterpart, the placebo effect. Patients’ experiences with healthcare are often dominated by negative emotions due to their association with illness and disease. Anxiety about health, fear, helplessness, and discomfort are common, especially in hospitals, where patients undergo diagnostic procedures that are unpleasant (Walker et al., 2021). This negative environment fosters nocebo effects, which are fueled by anxiety and negative emotions (Benson et al., 2023). The impact of nocebo effects is substantial, leading both to direct suffering from symptoms and indirect consequences such as patients refusing or discontinuing necessary diagnostic procedures or treatments due to fear of negative outcomes (Colloca and Miller, 2011).

This study protocol provides detailed documentation of the applied communication techniques and outcome measures to inform other scientists and clinical experts aiming to develop strategies to mitigate nocebo effects. As LPs are a common and indispensable component of diagnostics in neurology, a substantial number of patients could benefit from such research efforts. Moreover, the use of the optimized communication strategies for LP could serve as a showcase for other medical procedures and their respective complications, and this study protocol can inform the design of future trials. A further strength of this study protocol lies in its immediate translational potential for improving patient care by illustrating simple measures that can be readily incorporated into daily clinical routines. Evidence supporting the efficacy of optimized communication may lay the foundation for best practices in the endeavor to mitigate nocebo effects in healthcare. Such best practices could serve as a crucial guide for physicians experiencing the dilemma that arises from the motivation to uphold patient autonomy in shared decision-making while avoiding unintended harm to their patients by disclosing risks and informing them about necessary procedures or treatments. Detailed study protocols like the present one can inform educational programs directed at, for example, medical or nursing students and professional healthcare providers, an effort that has already been initiated (Ohlmann et al., 2024; Westendorp et al., 2024). Looking ahead, strategies for multicenter trials and the use of digital communication could help to improve communication on a broader scale (Meijers et al., 2023; Ponten et al., 2024).

A key limitation of the study is that it was not tailored to provide detailed insights into the psychoneurobiological factors underlying the effects of optimized communication, which are necessary for a more nuanced mechanistic understanding. These will need to be addressed in follow-up studies and include, for example, the role of fear, interrogation of symptom-specificity in side effect expectations, and investigation of potential endocrinological and neuroactivity correlates. Furthermore, a more granular profile of the patient’s personality could help identify those that benefit most from communication-related techniques to reduce nocebo effects. The data collected here will allow us to explore the influence of anxiety, depression, and illness perception on expectations and side effects. However, other traits such as suggestibility, neuroticism, or optimism—which others have shown to correlate with nocebo effects—have not been assessed here (Corsi et al., 2016; Corsi and Colloca, 2017; Karacaoglu et al., 2023). As our optimized ICP protocol combines both verbal and non-verbal strategies in one group, it does not allow us to directly isolate the specific contribution of verbal communication techniques or assess the added benefit of incorporating warm and empathetic non-verbal cues. Ultimately, studies that disentangle the contributions of each element of optimized communication to side effect outcomes in clinical cohorts will help identify the most effective strategies for different patient groups or symptoms.

## 4 Conclusion

This study protocol provides a comprehensive overview of the study design and intervention focused on enhancing communication during the informed consent process to reduce nocebo-related headaches. Conducting studies in real-world clinical environments is challenging but essential for translating insights from experimental nocebo studies into practical bedside applications. Our protocol is designed to serve as a guide for future studies.

## Footnotes

^1^ https://treatment-expectation.de/en/

^2^ https://www.R-project.org/
