# Supplementary material for: Optimized communication during risk disclosure to reduce nocebo headache after lumbar puncture—a study protocol for a randomized controlled clinical trial
Source: Front Psychol. 2025 Feb 26;16:1521978. doi: 10.3389/fpsyg.2025.1521978 (PMC11897036; doi:10.3389/fpsyg.2025.1521978)
Supplement: Supplementary file 1 [file Data_Sheet_1.zip › Supplementary Text S2.docx]

**Supplementary Text S2**

**Supplementary Text S2:** Text of the five modified items of the Treatment Expectation Questionnaire (TEX-Q) to assess expectations related to the lumbar puncture (LP).

Modified by Shedden-Mora and colleagues (Shedden-Mora, Alberts et al. 2023). The authors will provide the original German version upon request to the corresponding author.

The following questions concern what you personally think about your upcoming examination.

1. How much distress do you expect the procedure will cause? 0 = No distress; 10 = Greatest imaginable distress
2. To what extent do you expect side effects or other unwanted effects from the procedure? 0 = No unwanted effects; 10 = Greatest imaginable unwanted effects

The following questions concern what you expect from the course of your examination.

1. To what extent do you expect a pleasant course of treatment? 0 = Not pleasant; 10 = Most pleasant imaginable course
2. To what extent do you expect to be satisfied with the procedure? 0 = Not satisfied; 10 = Greatest imaginable satisfaction
3. To what extent do you expect your own behaviour to influence the success of the procedure? 0 = No responsibility; 10 = Greatest imaginable responsibility

References:

Shedden-Mora, M. C., J. Alberts, K. J. Petrie, J. A. C. Laferton, P. von Blanckenburg, S. Kohlmann, Y. Nestoriuc and B. Lowe (2023). "The Treatment Expectation Questionnaire (TEX-Q): Validation of a generic multidimensional scale measuring patients' treatment expectations." PLoS One **18**(1): e0280472.
