# Supplementary material for: Optimized communication during risk disclosure to reduce nocebo headache after lumbar puncture—a study protocol for a randomized controlled clinical trial
Source: Front Psychol. 2025 Feb 26;16:1521978. doi: 10.3389/fpsyg.2025.1521978 (PMC11897036; doi:10.3389/fpsyg.2025.1521978)
Supplement: Supplementary file 1 [file Data_Sheet_1.zip › Supplementary Videos S1 and S2.docx]

**Supplementary Videos 1 and 2**

Supplementary Videos 1 and 2 are available under:

<https://doi.org/10.6084/m9.figshare.28330721.v1>

DOI: 10.6084/m9.figshare.28330721

**Supplementary Video 1.**SOC.mp4: Demonstration of exemplary informed consent procedure according to the standard of care protocol (SOC) in English to demonstrate non-verbal cues. Please note that the English wording in the video can slightly differ from the final translations displayed in this publication.

**Supplementary Video 2.**OPT.mp4: Demonstration of informed consent procedure according to the optimized communication protocol (OPT) in English to demonstrate non-verbal cues. Please note that the English wording in the video can slightly differ from the final translations displayed in this publication.
