# Supplementary material for: Optimized communication during risk disclosure to reduce nocebo headache after lumbar puncture—a study protocol for a randomized controlled clinical trial
Source: Front Psychol. 2025 Feb 26;16:1521978. doi: 10.3389/fpsyg.2025.1521978 (PMC11897036; doi:10.3389/fpsyg.2025.1521978)
Supplement: Supplementary file 1 [file Data_Sheet_1.zip › Supplementary Table S1.docx]

**Supplementary Table S1.** SPIRIT 2013 and SPIRIT-PRO Extension Checklist: Recommended Items to Address in a Clinical Trial Protocol

This study protocol aligns with the SPIRIT guidelines (Standard Protocol Items: Recommendations for Interventional Trials) and follows the SPIRIT-PRO extension for patient-reported outcomes (Chan, Tetzlaff et al. 2013, Calvert, Kyte et al. 2018).

| **SPIRIT Section** | **SPIRIT Item No.** | **SPIRIT Item Description** | **SPIRIT-PRO Item No.** | **SPIRIT-PRO Extension** | **Addressed in section no.** |
| --- | --- | --- | --- | --- | --- |
| **Administrative Information** | | | | | |
| Title | 1 | Descriptive title identifying the study design, population, interventions, and, if applicable, trial acronym |  |  | See title page |
| Trial registration | 2a | Trial identifier and registry name(if not yet registered, name of intended registry) |  |  | See section 2.1 |
|  | 2b | All items from the World Health Organization Trial Registration Data Set |  |  | See section 2.1 |
| Protocol version | 3 | Date and version identifier |  |  | Version 2 (Dec 12, 2023) of the preregistration protocol at the German Clinical Trials Register (DRKS00032272) |
| Funding | 4 | Sources and types of financial, material, and other support |  |  | See Funding section |
| Roles and responsibilities | 5a | Names, affiliations, and roles of protocol contributors | SPIRIT- 5a-PRO Elaboration | Specify the individual(s) responsible for the PRO content of the trial protocol | Title page, author information, author contributions statement. Responsible for main outcome content: LA, DH, SB, UB |
|  | 5b | Name and contact information for the trial sponsor |  |  | The study is not sponsored |
|  | 5c | Role of study sponsor and funders, if any, in study design; collection, management, analysis, and interpretation of data; writing of the report; and the decision to submit the report for publication, including whether they will have ultimate authority over any of these activities |  |  | See Funding section. Funders had no role in any of the mentioned aspects. |
|  | 5d | Composition, roles, and responsibilities of the coordinating center, steering committee, end-point adjudication committee, data management team, and other individuals or groups overseeing the trial, if applicable (see item 21a for data monitoring committee) |  |  | There is no individual or group overseeing the trial. |
| **Introduction** | | | | | |
| Background and rationale | 6a | Description of research question and justification for undertaking the trial, including summary of relevant studies (published and unpublished) examining benefits and harms for each intervention | SPIRIT- 6a-PRO Extension | Describe the PRO-specific research question and rationale for PRO assessment and summarize PRO findings in relevant studies. | See sections 2.10 and 2.11 |
|  | 6b | Explanation for choice of comparators | SPIRIT-7-PRO Extension | State specific PRO objectives or hypotheses (including relevant PRO concepts/domains) | See sections 2.14.1 and 2.14.2 |
| Objectives | 7 | Specific objectives or hypotheses |  |  | See sections 2.14.1 and 2.14.2 |
| Trial design | 8 | Description of trial design including type of trial (eg, parallel group, crossover, factorial, single group), allocation ratio, and framework (eg, superiority, equivalence, noninferiority, exploratory) |  |  | See title page, and section 2.1 |
| **Methods** | | | | | |
| Study setting | 9 | Description of study settings (eg, community clinic, academic hospital) and list of countries where data will be collected. Reference to where list of study sites can be obtained |  |  | See sections 2.1 and 2.6 |
| Eligibility criteria | 10 | Inclusion and exclusion criteria for participants. If applicable, eligibility criteria for study centres and individuals who will perform the interventions (eg, surgeons, psychotherapists) | SPIRIT-10-PRO Extension | Specify any PRO-specific eligibility criteria (eg, language/reading requirements or prerandomization completion of PRO). If PROs will not be collected from the entire study sample, provide a rationale and describe the method for obtaining the PRO subsample. | See section 2.3 and 2.4 |
| Interventions | 11a | Interventions for each group with sufficient detail to allow replication, including how and when they will be administered |  |  | See section 2.8 |
|  | 11b | Criteria for discontinuing or modifying allocated interventions for a given trial participant (eg, drug dose change in response to harms, participant request, or improving/worsening disease) |  |  | See section 2.5 |
|  | 11c | Strategies to improve adherence to intervention protocols, and any procedures for monitoring adherence (eg, drug tablet return, laboratory tests) |  |  | See section 2.12 |
|  | 11d | Relevant concomitant care and interventions that are permitted or prohibited during the trial |  |  | No restrictions |
| Outcomes | 12 | Primary, secondary, and other outcomes, including the specific measurement variable (eg, systolic blood pressure), analysis metric (eg, change from baseline, final value, time to event), method of aggregation (eg, median, proportion), and time point for each outcome. Explanation of the clinical relevance of chosen efficacy and harm outcomes is strongly recommended | SPIRIT- 12-PRO Extension | Specify the PRO concepts/domains used to evaluate the intervention (eg, overall health-related quality of life, specific domain, specific symptom) and, for each one, the analysis metric (eg, change from baseline, final value, time to event) and the principal time point or period of interest. | See sections 2.10, 2.11, 2.12, |
| Participant timeline | 13 | Time schedule of enrolment, interventions (including any run-ins and washouts), assessments, and visits for participants. A schematic diagram is highly recommended |  |  | See section 2.12 and Figure 1 |
| Sample size | 14 | Estimated number of participants needed to achieve study objectives and how it was determined, including clinical and statistical assumptions supporting any sample size calculations |  |  | See section 2.7 |
| Recruitment | 15 | Strategies for achieving adequate participant enrolment to reach target sample size |  |  | See section 2.7 |
| **Methods: Assignment of Interventions** | | | | | |
| Allocation | | | | | |
| Sequence generation | 16a | Method of generating the allocation sequence (eg, computer-generated random numbers), and list of any factors for stratification. To reduce predictability of a random sequence, details of any planned restriction (eg, blocking) should be provided in a separate document that is unavailable to those who enrol participants or assign interventions |  |  | See section 2.7 |
| Allocation concealment mechanism | 16b | Mechanism of implementing the allocation sequence (eg, central telephone; sequentially numbered, opaque, sealed envelopes), describing any steps to conceal the sequence until interventions are assigned |  |  | See section 2.7 |
| Implementation | 16c | Who will generate the allocation sequence, who will enrol participants, and who will assign participants to interventions |  |  | See section 2.6 and 2.7 |
| Blinding (masking) | 17a | Who will be blinded after assignment to interventions (eg, trial participants, care providers, outcome assessors, data analysts) and how |  |  | See section 2.6 and 2.7 |
|  | 17b | If blinded, circumstances under which unblinding is permissible and procedure for revealing a participant’s allocated intervention during the trial |  |  | No such circumstances defined |
| Data collection methods | 18a | Plans for assessment and collection of outcome, baseline, and other trial data, including any related processes to promote data quality (eg, duplicate measurements, training of assessors) and a description of study instruments (eg, questionnaires, laboratory tests) along with their reliability and validity, if known. Reference to where data collection forms can be found, if not in the protocol |  |  | See section 2.12 |
|  | 18b | Plans to promote participant retention and complete follow-up, including list of any outcome data to be collected for participants who discontinue or deviate from intervention protocols |  |  | See section 2.5 |
| Data management | 19 | Plans for data entry, coding, security, and storage, including any related processes to promote data quality (eg, double data entry; range checks for data values). Reference to where details of data management procedures can be found, if not in the protocol |  |  | See section 2.13 |
| Statistical methods | 20a | Statistical methods for analysing primary and secondary outcomes. Reference to where other details of the statistical analysis plan can be found, if not in the protocol |  |  | See sections 2.14.1 and 2.14.2 |
|  | 20b | Methods for any additional analyses (eg, subgroup and adjusted analyses) |  |  | See section 2.14.3 |
|  | 20c | Definition of analysis population relating to protocol non-adherence (eg, as randomised analysis), and any statistical methods to handle missing data (eg, multiple imputation) |  |  | See section 2.5 |
| **Methods: Monitoring** | | | | | |
|  | 21a | Composition of data monitoring committee (DMC); summary of its role and reporting structure; statement of whether it is independent from the sponsor and competing interests; and reference to where further details about its charter can be found, if not in the protocol. Alternatively, an explanation of why a DMC is not needed |  |  | There is no data monitoring committee. |
|  | 21b | Description of any interim analyses and stopping guidelines, including who will have access to these interim results and make the final decision to terminatethe trial |  |  | No interim analyses |
| Harms | 22 | Plans for collecting, assessing, reporting, and managing solicited and spontaneously reported adverse events and other unintended effects of trial interventions or trial conduct |  |  | Patients were asked about unwanted side effects at all time points after the intervention as part of the study schedule. Patients were integrated in routine clinical care. See section 2.11 |
| Auditing | 23 | Frequency and procedures for auditing trial conduct, if any, and whether the process will be independent from investigators and the sponsor |  |  | No auditing trials are implemented. |
| **Ethics and Dissemination** | | | | | |
| Research ethics approval | 24 | Plans for seeking research ethics committee/institutional review board (REC/IRB) approval |  |  | See section 2.2 |
| Protocol amendments | 25 | Plans for communicating important protocol modifications (eg, changes to eligibility criteria, outcomes, analyses) to relevant parties (eg, investigators, REC/ IRBs, trial participants, trial registries, journals, regulators) |  |  | No modifications to the protocol have been implemented |
| Consent or assent | 26a | Who will obtain informed consent or assent from potential trial participants or authorised surrogates, and how (see Item 32) |  |  | See section 2.7 |
|  | 26b | Additional consent provisions for collection and use of participant data and biological specimens in ancillary studies, if applicable |  |  | See section 2.14 |
| Confidentiality | 27 | How personal information about potential and enrolled participants will be collected, shared, and maintained in order to protect confidentiality before, during, and after the trial |  |  | See sections 2.13 and 2.14 |
| Declaration of interests | 28 | Financial and other competing interests for principal investigators for the overall trial and each study site |  |  | See conflict of interest statement |
| Access to data | 29 | Statement of who will have access to the final trial dataset, and disclosure of contractual agreements that limit such access for investigators |  |  | See section 2.13 |
| Ancillary and posttrial care | 30 | Provisions, if any, for ancillary and post-trial care, and for compensation to those who suffer harm from trial participation |  |  | None specified |
| Dissemination policy | 31a | Plans for investigators and sponsor to communicate trial results to participants, healthcare professionals, the public, and other relevant groups (eg, via publication, reporting in results databases, or other data sharing arrangements), including any publication restrictions |  |  | Science communication to health care professionals and the public within the science communication of the SFB 289 "treatment expectation" (see section 2.13) |
|  | 31b | Authorship eligibility guidelines and any intended use of professional writers |  |  | See author contributions statement |
|  | 31c | Plans, if any, for granting public access to the full protocol, participant-level dataset, and statistical code |  |  | See section 2.13 |
| **Appendixes** | | | | | |
| Informed consent materials | 32 | Model consent form and other related documentation given to participants and authorised surrogates |  |  | Available upon request to the contributing author |
| Biological specimens | 33 | Plans for collection, laboratory evaluation, and storage of biological specimens for genetic or molecular analysis in the current trial and for future use in ancillary studies, if applicable |  |  | No biological specimens collected |

**References**

Calvert, M., D. Kyte, R. Mercieca-Bebber, A. Slade, A. W. Chan, M. T. King, S.-P. R. O. G. the, A. Hunn, A. Bottomley, A. Regnault, A. W. Chan, C. Ells, D. O'Connor, D. Revicki, D. Patrick, D. Altman, E. Basch, G. Velikova, G. Price, H. Draper, J. Blazeby, J. Scott, J. Coast, J. Norquist, J. Brown, K. Haywood, L. L. Johnson, L. Campbell, L. Frank, M. von Hildebrand, M. Brundage, M. Palmer, P. Kluetz, R. Stephens, R. M. Golub, S. Mitchell and T. Groves (2018). "Guidelines for Inclusion of Patient-Reported Outcomes in Clinical Trial Protocols: The SPIRIT-PRO Extension." JAMA **319**(5): 483-494.

Chan, A. W., J. M. Tetzlaff, P. C. Gotzsche, D. G. Altman, H. Mann, J. A. Berlin, K. Dickersin, A. Hrobjartsson, K. F. Schulz, W. R. Parulekar, K. Krleza-Jeric, A. Laupacis and D. Moher (2013). "SPIRIT 2013 explanation and elaboration: guidance for protocols of clinical trials." BMJ **346**: e7586.
