# Supplementary material for: Optimized communication during risk disclosure to reduce nocebo headache after lumbar puncture—a study protocol for a randomized controlled clinical trial
Source: Front Psychol. 2025 Feb 26;16:1521978. doi: 10.3389/fpsyg.2025.1521978 (PMC11897036; doi:10.3389/fpsyg.2025.1521978)
Supplement: Supplementary file 1 [file Data_Sheet_1.zip › Supplementary Text S1.docx]

**Supplementary Text S1**

**Supplementary Text S1:** Physician protocols for standard of care and optimized communication.

Context factors common to both groups: The same study physician carried out the informed consent procedure (ICP) and lumbar puncture (LP) for all patients in both groups. Clothing and equipment: closed white lab coat, glasses, work cellphone and ballpoint pens in the chest pocket of the lab coat, print information for LP, clipboard.

Control group: Standard of Care (SOC)

Non-verbal communication: Standing next to the patient, avoiding direct eye contact, arms mostly crossed, checking the time on the cellphone two or three times. No particular emphasis in voice modulation or facial expressions. Demeanor without engagement for further conversation, brief and concise responses to questions.

Guiding script for verbal information (translated from German):

*“Hello, so you’re Mrs…? Ahh, right.. Mrs XYZ. I’m Dr. XXX.*

*We have to perform a lumbar puncture on you, because we need the spinal fluid to proceed with your case. In order to be allowed to do this, I need to explain the procedure to you first before I then carry out the puncture.*

*[ explanation of individual indication for LP ]*

*The only way to access the spinal fluid is through a lumbar puncture. There isn’t a good alternative to investigating the spinal fluid, so unfortunately, there’s no way around it.*

*This is how it will go: In total, including preparation, it will take about 15 minutes. I will do it quickly right here on the edge of the bed. You'll sit there and arch your back. You need to tuck your chin into your chest. It’s important to stay in this position because otherwise, it will be more difficult for me to get the needle between the spinous processes of the vertebrae into the spinal canal. That’s where the spinal fluid is. As you can see, here in the spinal canal, where we want to go, is also the spinal cord. We want to avoid getting close to that at all cost. That’s why I will insert the needle lower down here. I will use an atraumatic needle to draw the spinal fluid. The tip of the needle may touch nerve fibers, which you might feel as a brief electric shock in your leg.*

*Unfortunately, complications cannot be completely ruled out. On this form here, various risks are listed. I will mention some of them to you. For example, infections are always possible when a needle is inserted into the body, as germs could potentially be introduced. Also, in principle, bleeding or tissue injury from the needle can technically occur, but this is very unlikely. We will drain some spinal fluid from the body. The most common side effect that could arise from this is headache. Therefore, you might get a headache, which is worse when you are upright, so sitting or standing. Such headaches occur in about 10 percent of cases after a lumbar puncture.*

*If headaches occur, you should lie flat and drink plenty of fluids, and if necessary, you can also get painkillers. We also recommend that you lie flat for one hour after the puncture and drink plenty of fluids. However, this is not strict bed rest. The individual results of the examination will be communicated to your doctor, who will share them with you.*

*Do you have any questions? If not, I would ask you to confirm your consent by signing here.*

*We can perform the lumbar puncture today; I’ll be able to squeeze it into my schedule, so you’ll get it over with quickly. I assume you don’t want to take 24 hours to think about it before we start the lumbar puncture?*

*So, I will come to you for the puncture at around xx o'clock.*

*Would you like a copy of this?”*

Experimental group: Optimized Communication (OPT)

Non-verbal communication: Sitting next to the patient, maintaining appropriate eye contact throughout, arms open, gesticulating where appropriate. Facial expressions and voice modulation altered through the conversation as appropriate. Demeanor: courteous, not interrupting; validating and understanding.

Guiding script for verbal information (translated from German):

*“Hello, it’s Mrs. Smith, right? My name is Dr. XXX, I am a doctor here in the neurology team. We’ve been advised to perform a lumbar puncture on you to examine the spinal fluid. Are you familiar with this procedure? I’ll start by giving you information and explaining everything to you, and I will later also be the person performing the procedure. We’re investigating the spinal fluid to better understand how we can best help you.*

*[ explanation of individual indication for LP ]*

*We frequently perform these spinal taps here. For me, it’s like drawing blood. We perform it on a daily basis. The spinal fluid is particularly helpful for understanding what’s happening in the nervous system. That's why we do it so often.*

*The whole process, including preparations, takes about 15 minutes. It will be done safely and comfortably here in your room. You’ll sit on the edge of the bed and arch your back. You can see an illustration of this here. It is important to tuck your chin into your chest as this is the ideal position. This will already help me a lot. Sometimes patients ask me whether the spinal cord isn’t close to that site. But I can reassure you, as that ends higher up and we are at a safe distance; our target area is down below here. Down here are the nerves for the legs, which float in the fluid like cooked spaghetti in water. It’s possible that we might tickle the nerve fibers during the procedure, which you might feel as a brief tingling in the leg. When that happens, we already know we’re in the right spot, and we’ll already be done after a few minutes.*

*As a doctor, it’s my duty to inform you about the risks of the procedure. However, side effects are rare, and the vast majority of patients don’t notice anything at all afterward. You should also know that sometimes side effects occur simply because you are informed about them. Just knowing about potential side effects can alter your body’s perception. It may have happened to you before, when you listen to yourself a bit more and become more aware of small changes that you wouldn’t otherwise notice. This phenomenon is called the nocebo effect, and it has been documented in many scientific studies. I can give you an example from my own experience: I was at a family gathering, and later it turned out that my nephews and nieces had headlice. You can’t imagine how much my head and everywhere else itched when I heard that, even though I didn’t have any lice myself in the end. So, my advice: Keep this in mind whenever you hear about side effects. It’s best to distract yourself after the procedure, perhaps by reading something.*

*I will work carefully and in sterile conditions to minimize the risk of infection. This is the most common procedure in neurology; we perform it several times a day at this university hospital, and I have never encountered a relevant bleed or injury caused by it. The body quickly replenishes the removed spinal fluid. Sometimes, patients may experience headache that improves significantly when lying down. However, the vast majority, about 9 out of 10 patients, don’t experience this at all.*

*If you do experience any such discomfort, you can help it pass quickly by simply lying flat and drinking plenty of fluids. We can also support you by giving you pain medication if necessary. We recommend that you lie flat for one hour after the procedure and drink plenty of fluids. However, this is not strict bed rest. You’ll most likely want to know when you’ll get the results—Dr. XYZ will discuss them with you as soon as they come back from the lab.*

*Do you have any questions? If not, I would ask you to confirm your consent by signing here.*

*We can perform the puncture today, so you’ll have the results quickly. Therefore, we’d like to offer to do this today.*

*I will come to you for the spinal tap at around X o'clock.*

*Would you like a copy of this?*

*Overall, it is a safe and very commonly performed routine procedure.”*

The authors will provide the original German guiding scripts upon request to the corresponding author.
